# Protective CD8^+^ T-cell response against Hantaan virus infection induced by immunization with designed linear multi-epitope peptides in HLA-A2.1/K^b^ transgenic mice

**DOI:** 10.1186/s12985-020-01421-y

**Published:** 2020-10-07

**Authors:** Ying Ma, Kang Tang, Yusi Zhang, Chunmei Zhang, Linfeng Cheng, Fanglin Zhang, Ran Zhuang, Boquan Jin, Yun Zhang

**Affiliations:** 1 Department of Immunology, The Fourth Military Medical University, 169 Changle West Road, Xi’an, 710032 China; 2 Department of Microbiology, The Fourth Military Medical University, Xi’an, 710032 China

**Keywords:** Hantaan virus, Multi-epitope peptide, Cytotoxic T cell response, Vaccine, HLA-A2.1/K^b^ transgenic mice

## Abstract

**Background:**

An effective vaccine that prevents disease caused by hantaviruses is a global public health priority, but up to now, no vaccine has been approved for worldwide use. Therefore, novel vaccines with high prophylaxis efficacy are urgently needed.

**Methods:**

Herein, we designed and synthesized Hantaan virus (HTNV) linear multi-epitope peptide consisting of HLA-A*02-restricted HTNV cytotoxic T cell (CTL) epitope and pan HLA-DR-binding epitope (PADRE), and evaluated the immunogenicity, as well as effectiveness, of multi-epitope peptides in HLA-A2.1/K^b^ transgenic mice with interferon (IFN)-γ enzyme-linked immunospot assay, cytotoxic mediator detection, proliferation assay and HTNV-challenge test.

**Results:**

The results showed that a much higher frequency of specific IFN-γ-secreting CTLs, high levels of granzyme B production, and a strong proliferation capacity of specific CTLs were observed in splenocytes of mice immunized with multi-epitope peptide than in those of a single CTL epitope. Moreover, pre-immunization of multi-epitope peptide could reduce the levels of HTNV RNA loads in the liver, spleen and kidneys of mice, indicating that specific CTL responses induced by multi-epitope peptide could reduce HTNV RNA loads in vivo.

**Conclusions:**

This study may provide an important foundation for the development of novel peptide vaccines for HTNV prophylaxis.

## Background

Rodent-borne orthohantaviruses of the family *Hantaviridae* are widespread zoonotic pathogens that cause two diseases in humans, hemorrhagic fever with renal syndrome (HFRS) and hantavirus cardiopulmonary syndrome (HCPS) [1, 2]. At present, over 28 hantaviruses have been identified. The Old World hantaviruses, such as the prototypic member Hantaan virus (HTNV), Puumala virus (PUUV) and Dobrava virus (DOBV), are etiological agents in Asia and Europe, and together with the worldwide distributed Seoul virus (SEOV), they cause the majority of cases of HFRS [3, 4]. While New World hantaviruses, including the Andes virus (ANDV) and Sin Nombre virus (SNV), were found predominantly in the Americas—causing HCPS with a fatality rate of up to 40% of cases [5]. Hantaviruses are maintained in rodent reservoirs and are usually transmitted to humans in aerosols of rodent excreta, causing syndromes ranging from acute renal failure to pulmonary oedema and severe hemorrhagic illness [3]. So far, around 1000 HCPS cases have been reported, whereas more than 100,000 HFRS cases are estimated to occur worldwide each year [3]. China has the largest number of HFRS infections, with a fatality rate of up to 15% of cases, which accounted for nearly 90% of the global HFRS cases [6, 7]. Regarding their impacts on public health, hantaviruses-associated diseases merit significantly more attention from researchers and officials, and are considered to be a high-priority public health concern in many countries [8].

Approved HFRS inactivated vaccines are a reality in China and Korea [9]. Approximately 2 million doses of inactivated rodent brain or cell-culture-derived HFRS vaccines are administered annually in China [10, 11]. Although vaccination, along with other preventative measures, has coincided with a reduction in HFRS cases in these regions, the inactivated vaccines fails to demonstrate a statistically significant reduction in the disease severity of HFRS [12]. Currently, there are no U.S. Food and Drug Administration (FDA)-approved vaccines or treatments for these hantavirus diseases [10]. The World Health Organization-approved HFRS vaccines also remain unavailable. One of the new strategies for innovative vaccine development is a multi-epitope-based peptide vaccine, which has been under study in many infectious diseases and cancers, and has even progressed to preclinical or clinical trials [13–15]. A multi-epitope-based peptide vaccine offering the capability of eliciting both cell-mediated responses and antibody (Ab) responses has several advantages, such as being easily produced, chemically stable and free of bacterial/viral contaminating substances [16]. Thus, the identification of the epitopes, especially the immunoprotective epitopes, could provide a basis for the development of epitope-based peptide vaccines. However, there are hardly any studies on the design and evaluation of the multi-epitope-based peptide vaccine for HFRS infected with HTNV.

The antigenicity of hantaviruses largely depends upon two structural proteins, nucleocapsid protein (NP) and envelope glycoprotein (GP), encoded by the negative-sense RNA genome. Both NP and GP, heterodimers of mature glycoprotein Gn and Gc, have been confirmed to be responsible for vigorous cellular and humoral immune responses [12, 13]. Several studies have demonstrated that both vigorous cytotoxic T cell (CTL) responses and multifunctional T helper (Th) cell responses are important for protection against HTNV infection [17–19]. In our previous study, eight CTL epitopes on HTNV-NP with different HLA restrictions and seven CTL epitopes on HTNV-GP-restricted by HLA-A*02 have been identified, meanwhile several CD4^+^ T-cell epitopes from HTNV-GP were also defined [20–23]. Importantly, these epitopes have been confirmed for the induction of immunoprotective responses in HFRS patients or in mice, which exactly provided the basis to develop an epitope-based vaccine for HFRS [20–23].

Notably, the specific CD4^+^ T-cells could exert the effects further through the stimulation of B-cells for neutralizing antibody production and promoting the CD8^+^ T-cell response during infections. Thus, CD4^+^ T-cell epitopes may be of great importance in the design of multi-epitope peptide vaccines. The pan HLA-DR binding epitope (PADRE), which can bind various MHC class II molecules with high affinity, is capable of generating antigen-specific CD4^+^ T-cells and has been widely used to construct the multi-epitope peptide vaccines [24]. Several epitope-based vaccines for diseases such as HIV, cytomegalovirus infection and hepatocellular carcinoma, represented T-cell incorporated alongside a universal PADRE to improve the potency of their immune-response stimulation [25–28]. Therefore, we propose that PADRE peptide may provide a powerful immunogen in the development of multi-epitope peptide vaccines of HTNV.

In this study, HTNV linear multi-epitope peptide, composed of single HLA-A*02-restricted HTNV CTL epitope and PADRE were designed and constructed as candidate vaccine. Moreover, specific CD8^+^ T-cells responses and characteristics induced by the multi-epitope peptide were analyzed in HLA-A2.1/K^b^ transgenic (Tg) mice to evaluate the immunogenicity and protective effects of the peptide, which may provide some important information for the development of a novel peptide vaccine for HFRS infected by HTNV.

## Methods

### Mice

HLA-A2.1/K^b^ transgenic (Tg) mice, which were originally purchased from the Jackson Laboratory (BarHarbor, ME), were kindly provided by Dr. Yuzhang Wu (Third Military Medical University, Chongqing, China). All Tg mice were maintained under pathogen-free conditions with good welfare in the Animal House Facility at the Center for Laboratory Animal, Fourth Military Medical University, Xi’an, China. A chimeric gene, consisting of the α1 and α2 domains of HLA-A*0201 and the α3 transmembrane and cytoplasmic domain of H-2K^b^, was represented in the Tg mice with C57BL/6 background. HLA-A*02 expression on the cell surface was detected by phycoerythrin (PE)-labelled anti-HLA-A*02 mAb BB7.2 (Biolegend, San Diego, CA, USA) in flow cytometry.

### Peptide design and synthesis

Based on our previous study, the identified HLA-A*02 restricted HTNV-GP CD8^+^ T-cell epitope (aa8-aa16, VMASLVWPV, VV9) were selected to construct multi-epitope peptides, given that epitope VV9 has been proven to bind with HLA-A*02 with highest affinity among the seven HLA-A*02-restricted HTNV-GP CTL epitope [23]. The Pan HLA-DR T-helper epitope (PADRE, AKXVAAWTLKAAA, X = cyclohexylalanine) [29] was used to connect the HTNV epitopes in series. The linear multi-epitope peptide consists of the HLA-A*02-restricted HTNV CTL epitope (VV9) in tandem with PADRE, defined as “PADRE-VV9”. The K-S-S was used as the adaptor sequence and the A-A-A in PADRE could be used as the linker sequence for the connection of the multi-epitope peptide. All the peptides were synthesized and characterized by analytical high-performance liquid chromatography (HPLC) and mass spectrometry by Synpeptide. Co. Ltd (Nanjing, China), with purity > 95%. Lyophilized peptides were resuspended in sterile phosphate-buffered saline (PBS) solution at 1 mM and stored in aliquots at − 80 °C until further use.

### Vaccine and Virus

The commercial bivalent and purified HFRS inactivated vaccine (YOUERJIAN®, Zhejiang Tianyuan Bio- Pharmaceutical Co., Ltd., China) composed of a mixture of both HTNV and SEOV was provided as a positive control in the immunization of the Tg mice. The HTNV 76-118 strain was preserved and kindly provided by the Department of Microbiology of the Fourth Military Medical University.

### Immunization of HLA-A2.1/K^b^ Tg mice with the peptide

Fifty male six-to-eight-week-old HLA-A2.1/K^b^ Tg mice were divided into five groups (n = 10 each) for immunization, including the single HTNV CTL epitope VV9 immunization group, HTNV linear multi-epitope peptide PADRE-VV9 immunization group, unrelated peptide HLA-B*35-restricted HTNV CTL epitope (VPILLKALY, VY9) immunization group [21], inactivated vaccine inoculation group and PBS injection group. The peptide immunizations of the Tg mice were carried out using an N-terminal fragment of the murine glycoprotein 96 (gp96) as a chaperone [30, 31]. The heat shock protein gp96 and its N-terminal fragment (N333) showed adjuvant effects in enhancing the peptide-specific CTL response through binding with peptide. The association of gp96 with MHC ligands may indicate its role in MHC class I peptide processing and presentation [32]. Meanwhile, complete/incomplete Freund's adjuvants were also used for Tg mice immunization. The *Mycobacteria* in complete Freund′s adjuvant could attract macrophages and other cells to the injection site, which enhances the immune response. Therefore, complete Freund′s adjuvant is used for the initial injections. While incomplete Freund′s adjuvant, which lacks the *Mycobacteria*, is used for subsequent boost injections.

Briefly, for the first-time immunization, 50 μg peptide was mixed with 30 μg of the N-terminal fragment N333 (aa22-aa355) of murine gp96. Then, the mixture was emulsified in 1:1 ratio with complete Freund’s adjuvant (Difco), and was adjusted to 100 μL of the injection volume for each Tg mouse. The immunization was performed via subcutaneous injection at 4–5 locations on the neck and back of each mouse. The dose of each injection location was about 20–25 μL. The same immunization methods were carried out for the second- and third-round immunization of the Tg mice with the peptide and gp96 mixture emulsified in 1:1 ratio with incomplete Freund’s adjuvant (Difco). Each peptide was prepared separately using the same method. Replacing the peptide with PBS, and then immunizing Tg mice with the same method, was used as a negative control group. Subcutaneous injections with the commercialized HFRS inactivated vaccine were used as the positive control groups. Specifically, 10 μL of the injection volume of the commercialized HFRS inactivated vaccine was used for each round immunization in each Tg mouse [33]. The inactivated vaccine immunization was performed via subcutaneous injection at one location on the neck of each mouse. The same method was then used for the second- and third-round immunization of inactivated vaccine in Tg mice. All Tg mice were injected the same number of times. Three immunization injections were administered to each mouse at intervals of two weeks. Ten days after the last immunization, half number of the mice (n = 5) in each immunized group was euthanised by cervical vertebra dislocated method. The splenocytes were isolated from each mouse using lymphocyte separation medium (Dakewe Biotech Company Ltd, Shenzhen, China).

### HTNV challenge

The remaining immunized HLA-A2.1/K^b^ Tg mice (n = 5) in each group were subsequently challenged with intramuscular injection of the HTNV 76-118 strain (1 × 10^5^ pfu/mouse) ten days after the final immunization booster. On day 4 post-challenge, the Tg mice were sacrificed. Tissue samples from six major organs, including cerebrum, heart, liver, spleen, lung and kidney, were harvested for viral load detection.

### Determination of relative HTNV RNA loads with quantitative RT-PCR

The tissue samples of the six major organs from post-exposure Tg mice were preserved in a non-frozen tissue RNA preservation solution (Solarbio, CN) at − 4 °C. An RNA purification extraction kit (Tiangen Biotech, CN) was used to extract the tissue RNA of each type of organ. The extracted tissue RNA was then used as a template for reverse transcription to obtain the cDNA with PrimeScript™ RT-PCR kit (Takara). The target sequence of the HTNV S segment was detected for determination of the relative HTNV RNA loads in each organ sample using an SYBR real-time quantification PCR kit (Takara) with the following primers: HTNV forward, 5′-GATCAGTCACAGTCTAGTCA-3′; HTNV reverse, 5′-TGATTCTTCCACCATTTTGT-3′; mouse GAPDH forward, 5′-AGGCCGGTGCTGAGTATGTC-3′; mouse GAPDH reverse, 5′-TGCCTGCTTCACCACCTTCT-3′. The results were recorded as cycle time (*Ct*) and quantified by 2^−ΔΔCt^.

### Ex vivo IFN-γ enzyme-linked immunospot (ELISPOT) assay

The IFN-γ ELISPOT assay (Dakewe Biotech Company Ltd, Shenzhen, China) was used for the determination of the immunogenicity of the HTNV linear multi-epitope peptide ex vivo [21]. Briefly, the splenocytes isolated from immunized HLA-A2.1/K^b^ Tg mice in each group were placed on ELISPOT plates (IFN-γ mAb precoated), at 1 × 10^6^ cells/well. Cells were stimulated with the single HTNV CTL epitope (10 μM) for 24 h at 37 °C. Phytohemagglutinin (PHA, 10 μg/mL, Sigma-Aldrich, St. Louis, MO, USA) stimulation served as a positive control, while the irrelevant peptide and media alone served as negative controls. Spots developed after incubation for 10–30 min with 3-amino-9-ethylcarbazole (AEC) substrate in the dark and counted with an ELISPOT plate reader (Cellular Technology Limited, USA). The number of spots was expressed as adjusted spot-forming cells (SFC)/10^6^ splenocytes after subtracting the average negative values. The positive response was defined as at least 50 SFC/10^6^ input cells, exceeding 3-times the background response. The SFC/10^6^ splenocytes in the unstimulated control wells never exceeded 5 spots per well.

### Intracellular cytokine staining and degranulation assay

Splenocytes (2 × 10^6^) obtained from the immunized HLA-A2.1/K^b^ Tg mice were restimulated with HTNV single CTL epitope (5 μM) in the presence of 1 μg/mL costimulatory molecules anti-mouse CD49d mAb (clone 9C10) and anti-mouse CD28 mAb (clone 37.51) (Biolegend, San Diego, CA, USA). One hour after peptide stimulation, 10 μg/mL brefeldin A (Sigma-Aldrich, St. Louis, MO, USA) and 0.7 μl/mL monensin (Golgistop, BD Biosciences, San Jose, CA, USA) were added, with an additional 5 h incubation at 37 °C. Cells stimulated with 0.1 μg/mL phorbol myristate acetate (PMA, Sigma-Aldrich, St. Louis, MO, USA) and 0.05 μg/mL ionomycin (Sigma-Aldrich, St. Louis, MO, USA) or medium alone were used as positive or negative controls, respectively. The cells were washed and stained for surface markers with anti-mouse CD3-PerCP-Cy5.5 (clone 17A2) and anti-mouse CD8α-APC (clone 53-6.7) mAbs (Biolegend, San Diego, CA, USA), fixed and permeabilized using a Cytofix/Cytoperm™ Plus Fixation/Permeabilization Kit (BD Pharmingen, San Diego, CA, USA), and then stained with anti-mouse granzyme B-FITC mAb (clone GB11) (Biolegend, San Diego, CA, USA). FITC-, PerCP-Cy5.5- and APC-conjugated mouse IgG1, κ were used as isotype controls. A total of 200,000 events per sample were collected using FACSCalibur™ (BD Biosciences, San Jose, CA, USA). The analysis was performed immediately with FlowJo version 9.2 (TreeStar, Ashland, OR, USA). Splenocytes were defined as FSC/SSC, and CD8^+^ T-cells were defined as CD3^+^CD8^+^ events, displayed on a dot plot of CD8 versus granzyme B. The positive response was determined as being when the percentage of cytokine was greater than 0.1% after background subtraction.

### Ex vivo proliferation assay

Splenocytes (2 × 10^7^/mL) from the immunized Tg mice were labeled with 10 μM 5,6-carboxyfluorescein succinimidyl ester (CFSE, Molecular Probes, Eugene, OR, USA) at 37 °C for 15 min, and terminated upon the addition of equivoluminal fetal bovine serum without dilution. Then, the CFSE-labeled cells were stimulated with HTNV single CTL epitope (10 μM). PHA (10 μg/mL, Sigma-Aldrich, St. Louis, MO, USA) stimulation served as a positive control. Cells without stimulation were used as background control. After 2 days, 10% exogenous IL-2 (20 U/mL) was added. After 7 days from the peptide stimulation, the cells were harvested and stained with the anti-mouse CD3-PerCP-Cy5.5 (clone 17A2), anti-mouse CD8-APC (clone 53-6.7) mAbs (Biolegend, San Diego, CA, USA). Approximately 300,000 cells were acquired using a FACSCalibur™ (BD Biosciences, San Jose, CA, USA).

### Statistical analysis

Statistical analysis was performed using SPSS 16.0 (SPSS Inc., Chicago, IL, USA) and graphing was performed using GraphPad Prism software, version 6 (GraphPad; La Jolla, CA, USA). The frequency of the CD8^+^ T-cells and the cytokines secreted are presented as the mean ± standard error of mean (SEM). The unpaired *t* test was used for comparison of the parameters between the two subject groups. A *P* value below 0.05 (*P* ≤ 0.05) was considered to be statistically significant.

### Accession numbers

HTNV 76-118 strain glycoprotein GenBank accession number: P08668.1; HTNV 76-118 strain nucleoprotein GenBank accession number: M14626.

### Ethics approval

The research was conducted in strict accordance with the recommendations in the Guide for the Care and Use of Laboratory Animals of the National Health and Medical Research Council of China. The protocol was approved by the Committee on the Ethics of Animal Experiments of the Fourth Military Medical University with the license number XJYYLL-2014437. All procedures were performed under sodium pentobarbital anesthesia, with the best effort to minimize animal suffering.

## Results

### Construct and synthesis of linear HTNV multi-epitope peptide

**Fig. 1:**
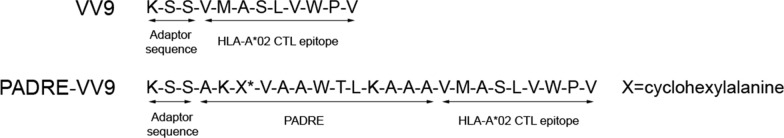
Details of the primary sequences of Hantaan virus (HTNV) linear multi-epitope peptides. The *top row* shows the HLA-A*02-restricted single HTNV-GP CD8^+^ T-cell epitope with the amino acid sequence VMASLVWPV defined as “VV9”. The *bottom row* shows the HTNV linear multi-epitope peptide, consisting of the same HLA-A*0201-restricted HTNV CTL epitope VV9 in tandem with Pan HLA-DR T helper epitope (PADRE, AKXVAAWTLKAAA, X = cyclohexylalanine), defined as “PADRE-VV9”. The K-S-S was used as the adaptor sequence and the A-A-A in PADRE was used as the linker sequence for the multi-epitope peptides connection. CTL, cytotoxic T lymphocyte. X* indicates cyclohexylalanine

Based on our previous findings, the immunoprotective HLA-A*02-restricted HTNV-GP 9-mer CTL epitope VV9 and the universal T-helper epitope PADRE were selected to design linear multi-epitope peptide as candidate vaccine for HTNV. The linear peptide possessed PADRE with VV9 epitope and was referred to as “PADRE-VV9”. The primary sequences of the peptide immunogen are shown in Fig. 1.

### The strong capacity of linear multi-epitope peptide to induce HTNV-specific IFN-γ-secreting CD8^+^ T-cell responses in HLA-A2.1/K^b^ Tg mice

**Fig. 2:**
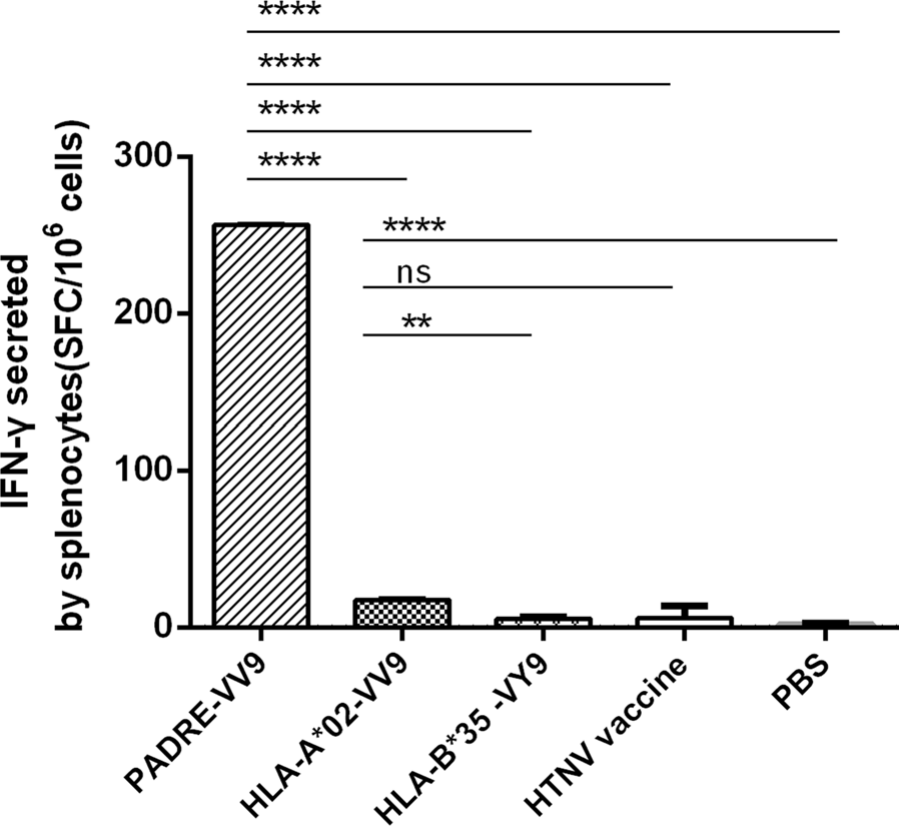
Analysis of HTNV-specific IFN-γ-secreting CD8^+^T-cell responses of HLA-A2.1/K^b^ Tg mice after immunization by different peptides. Comparison of the frequencies (*y-axis*) of IFN-γ-secreting CD8^+^ T-cells in ex vivo ELISPOT assay among the different immunization groups (*x-axis*). The Tg mice were divided into five groups according to the different immunization, including HTNV linear multi-epitope peptide PADRE-VV9, HLA-A*02-restricted single HTNV-GP CTL epitope VV9, HLA-B*35-restricted HTNV-NP CTL epitope VY9 as an unrelated peptide control, HTNV-inactivated vaccine as a positive control and PBS as a negative control, respectively. The frequencies of the IFN-γ secreting CD8^+^ T-cell responses are represented as the SFC/10^6^ splenocytes. The unpaired *t* test was used for statistical evaluation. IFN, interferon. SFC, spot-forming cells. PBS, phosphate buffer saline. VV9, VMASLVWPV. VY9, VPILLKALY. PADRE, AKXVAAWTLKAAA, X = cyclohexylalanine. ****P < 0.0001; **P < 0.01. ns, not significant

The humanized HLA-A2.1/K^b^ Tg mice were employed for peptide immunization. Splenocytes from primed Tg mice were tested for IFN-γ production in ELISPOT assay for validation of the ability to elicit specific CD8^+^ T-cell responses. The results showed that the mean ± SEM frequency of IFN-γ-secreting CD8^+^ T-cells from Tg mice inoculated with the single HTNV CTL epitope VV9 was 17.33 ± 0.67 SFC/10^6^ splenocytes. Whereas high frequency of IFN-γ production CD8^+^ T-cells in multi-epitope peptide PADRE-VV9-immunized group was observed with a mean ± SEM magnitude of 256.50 ± 0.50 SFC/10^6^ splenocytes, which was significantly higher than that in the VV9-immunized group (*P* < 0.0001), unrelated HLA-B*35-restricted VY9 peptide-immunized group (mean ± SEM, 5.50 ± 1.50 SFC/10^6^ splenocytes, *P* < 0.0001) and PBS-injection group (mean ± SEM, 1.67 ± 0.67 SFC/10^6^ splenocytes, *P* < 0.0001) (Fig. 2). PADRE-VV9-immunized group could induce an even higher frequency of IFN-γ-secreting CD8^+^ T-cells than that in the HTNV-inactivated vaccine positive control group (mean ± SEM, 6.00 ± 4.50 SFC/10^6^ splenocytes, *P* < 0.0001) (Fig. 2).

### The cytolytic mediators production from specific CD8^+^ T-cells in HLA-A2.1/K^b^ Tg mice immunized with HTNV linear multi-epitope peptide

**Fig. 3:**
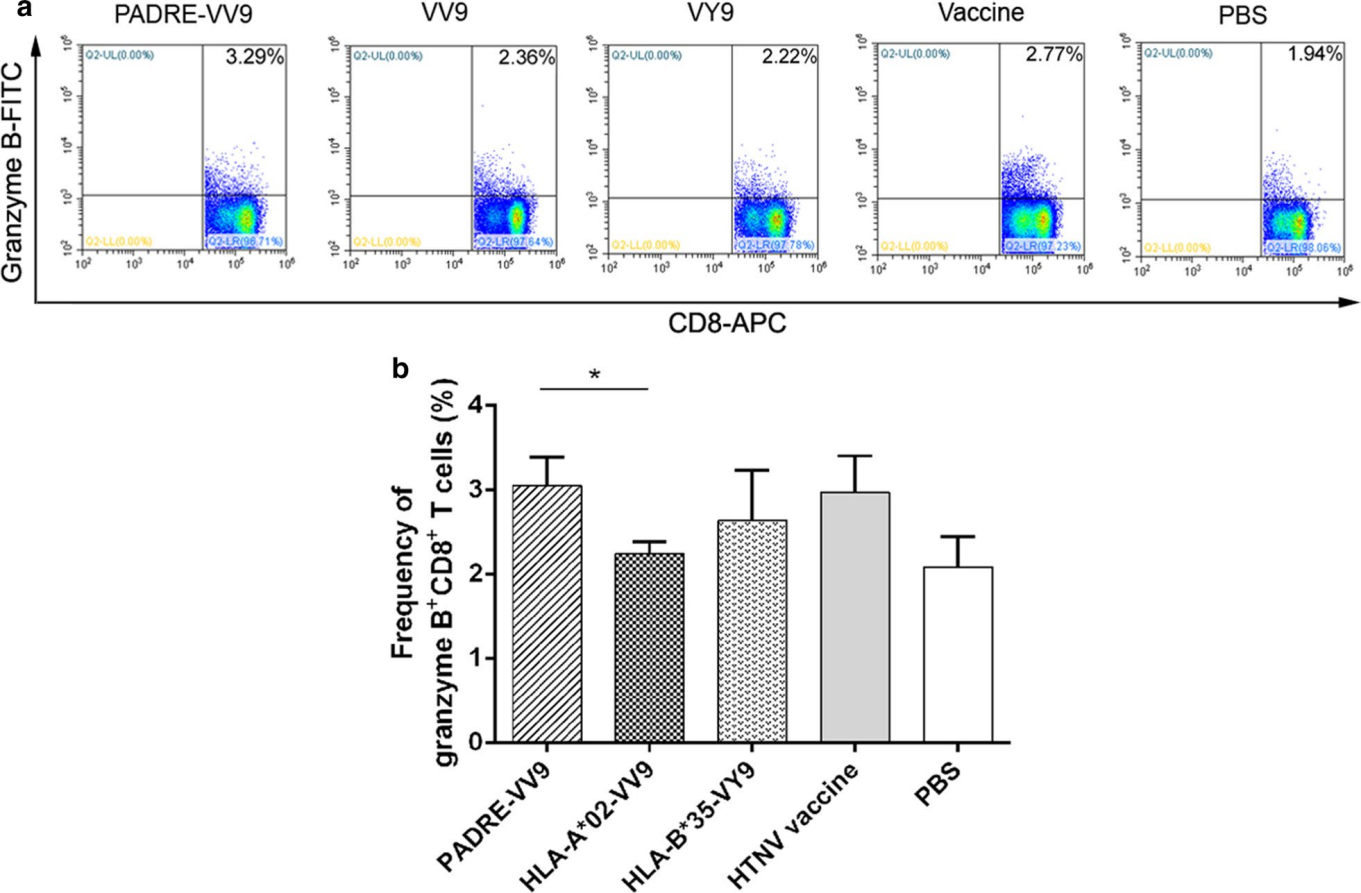
The production of cytolytic mediators of HTNV-specific CD8^+^T-cells in HLA-A2.1/K^b^ Tg mice after peptide immunization. The Tg mice were divided into five groups, including the mice immunized with HTNV linear multi-epitope peptide PADRE-VV9, HLA-A*02-restricted single HTNV-GP CTL epitope VV9, HLA-B*35-restricted HTNV-NP CTL epitope VY9, HTNV-inactivated vaccine and PBS injection, respectively. **a** Representative flow cytometric plots of cytotoxic mediator granzyme B-producing CD8^+^ T-cells in splenocytes of the Tg mice after three immunizations in different groups. The numbers indicate the percentage of cells within the boxed regions. **b** Comparison of the percentage of granzyme B^+^ CD8^+^ T-cells (*y-axis*) among the five different mice groups (*x-axis*). Peptide VY9-immunized mice were used as an unrelated peptide control, HTNV-inactivated vaccine-immunized mice were used as a positive control and PBS-administered mice were used as a negative control, respectively. The bar in each group represents the mean value ± standard error of mean (SEM). For the gating strategy, splenocytes were defined as FSC/SSC, and CD8^+^ T-cells were defined as CD3^+^CD8^+^ events, displayed on a dot plot of CD8 versus granzyme B. The unpaired *t* test was used for statistical evaluation. PBS, phosphate buffer saline. VV9, VMASLVWPV. VY9, VPILLKALY. PADRE, AKXVAAWTLKAAA, X = cyclohexylalanine. *P < 0.05

The cytotoxic capacity of specific CD8^+^ T-cells producing granzyme B was evaluated with intracellular staining assay in different immunized or treatment Tg mice groups. Both HTNV single epitope and linear multi-epitope peptide could induce the CD8^+^ T-cells of splenocytes in Tg mice to produce granzyme B at different levels after immunization (Fig. 3a). Notably, the frequency of granzyme B^+^ CD8^+^ T-cells in the PADRE-VV9-immunized group (mean ± SEM, 3.06 ± 0.24) was higher than that in the single CTL epitope-immunized group (mean ± SEM, 2.24 ± 0.08, *P* = 0.029) (Fig. 3b), indicating the strong capacity of the HTNV multi-epitope peptide to induce effective CTL responses. Moreover, there was no difference of granzyme B^+^ CD8^+^ T-cells frequency between the single CTL epitope-immunized group and the PBS group ( mean ± SEM, 2.09 ± 0.21, *P* = 0.526).

### The ex vivo specific CD8^+^ T-cell expansion stimulated with HTNV linear multi-epitope peptide in HLA-A2.1/K^b^ Tg mice

**Fig. 4:**
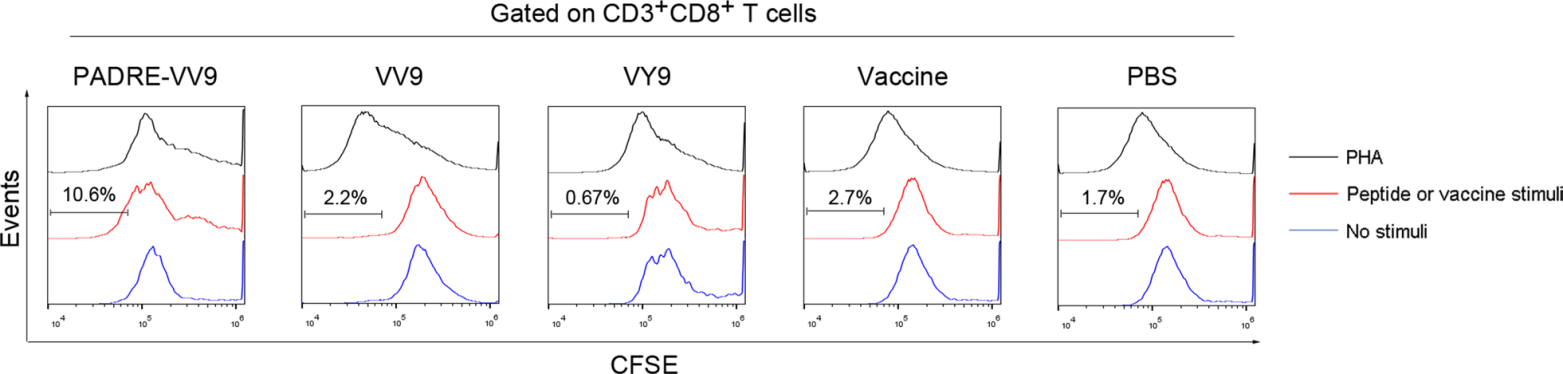
The proliferation of HTNV-specific CD8^+^T-cells stimulated by peptide in HLA-A2.1/K^b^ Tg mice with different immunization. Representative flow cytometric histograms of the expansion percentage of HTNV-specific CD8^+^ T-cells stimulated by peptides in each immunized Tg mice group, including immunization with HTNV linear multi-epitope peptide PADRE-VV9, HLA-A*02-restricted single HTNV-GP CTL epitope VV9, HLA-B*35-restricted HTNV-NP CTL epitope VY9 (unrelated peptide control), the HTNV-inactivated vaccine (positive control) and PBS injection (negative control), respectively. Each group containing three conditions in histograms clearly demonstrated that the extent of CFSE curve shifted to the left represented the percentage of CD8^+^ T-cell proliferation. The *middle row* shows the epitope VV9 stimulation. The *upper* and the *lower rows* showed the PHA stimulation as positive control and no stimuli as negative control, respectively. The analysis of the cells was carried out using gated CD3^+^CD8^+^ T-cells. CFSE, 5, 6-carboxyfluorescein succinimidyl ester. PHA, phytohemagglutinin. PBS, phosphate buffer saline. VV9, VMASLVWPV. VY9, VPILLKALY. PADRE, AKXVAAWTLKAAA, X = cyclohexylalanine

We next assessed whether the HTNV linear multi-epitope peptide could enhance the proliferation of CD8^+^ T-cells in Tg mice after immunization. CFSE was used for cell proliferation assays. The results showed that the epitope VV9-specific CD8^+^ T-cells were expanded from Tg mice immunized with either HTNV single CTL epitope or linear multi-epitope peptide (Fig. 4). There was a wider peak with two small peaks observed in the results of CSFE staining of the PADRE-VV9-immunized mice stimulated by the peptide. The peak at the left side referred to the proliferated cells. Specifically, there was 2.2% proliferation of the specific CD8^+^ T-cells in the single CTL epitope VV9-immunized group (Fig. 4). While PADRE-VV9-immunized groups yielded 10.6% proliferation of specific CD8^+^ T-cells, which was also higher than that in the unrelated peptide VY9-immunized group (0.67%), the inactivated vaccine group (2.7%) and PBS-injection group (1.7%) (Fig. 4).

### Immunization of HTNV linear multi-epitope peptide reduced HTNV RNA loads in HLA-A2.1/K^b^ Tg mice after HTNV challenge

**Fig. 5:**
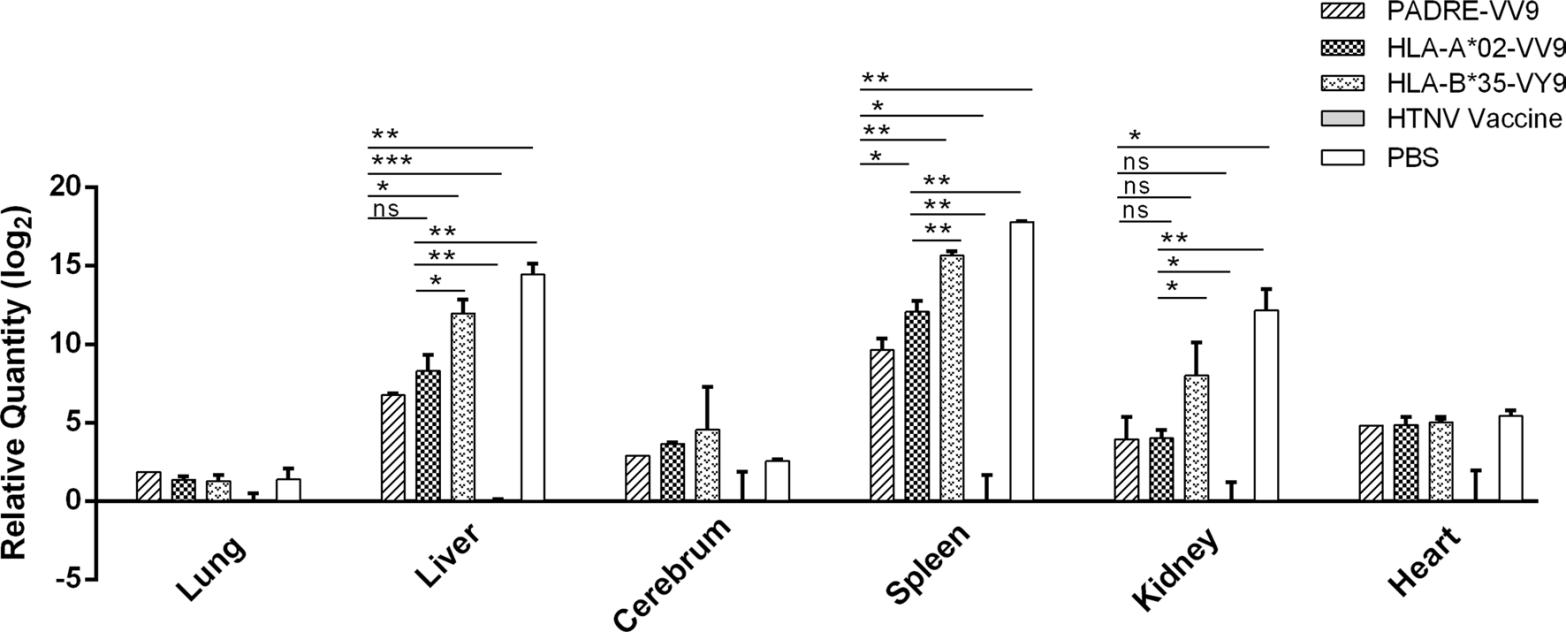
Detection of HTNV RNA loads in organs of HTNV-challenged HLA-A2.1/K^b^ Tg mice after peptide immunization. The HLA-A2.1/K^b^ Tg mice were divided into five groups, immunized with HTNV linear multi-epitope peptide PADRE-VV9, HLA-A*02-restricted single HTNV-GP CTL epitope VV9, HLA-B*35-restricted HTNV-NP CTL epitope VY9 as an unrelated peptide control, the HTNV-inactivated vaccine as a positive control and PBS as a negative control, respectively. The HTNV RNA loads (*y-axis*) were detected via real-time PCR in the lungs, liver, cerebrum, spleen, kidneys and heart (*x-axis*) of Tg mice after HTNV challenge in the five different immunization groups. Comparison of the HTNV RNA loads which could be detected in liver, spleen and kidneys of the immunized Tg mice after HTNV challenge among five immunization mice groups, respectively. The results shown were recorded as cycle time (*Ct*) and quantified by 2^−ΔΔCt^. The bar in each group represents the mean value ± standard error of mean (SEM). The unpaired *t* test was used for statistical evaluation. PBS, phosphate buffer saline. VV9, VMASLVWPV. VY9, VPILLKALY. PADRE, AKXVAAWTLKAAA, X = cyclohexylalanine. ***P < 0.001; **P < 0.01; *P < 0.05. ns, not significant

Next, the HTNV challenge experiments were conducted to determine whether HTNV linear multi-epitope peptide immunization could defend against HTNV infection in vivo. A model of HTNV infection and replication in HLA-A2.1/K^b^ Tg mice was established from a previous study [20, 23]. Consistent with the previous detection, few detectable HTNV RNA could be observed in the lungs, cerebrum and heart in all Tg mice groups after HTNV challenge, whereas high levels of HTNV RNA loads were displayed in the liver, spleen and kidney in each Tg mouse from the negative control group injected with PBS, suggesting that the liver, spleen and kidney are the major organs for HTNV infection and replication in Tg mice. As expected, a significantly low level of the HTNV RNA loads was observed in Tg mice immunized with HTNV-inactivated vaccine after HTNV challenge. The mice immunized with the irrelevant control peptide HTNV-NP VY9 showed markedly higher levels of HTNV RNA loads in the liver, spleen and kidneys (Fig. 5).

In the Tg mice group immunized with HLA-A*02-restricted single HTNV CTL epitope VV9, the viral loads were reduced in the liver, spleen and kidneys, which were lower than that in the PBS-injection negative control group (*P* = 0.0057 in the liver, *P* = 0.0017 in the spleen and *P* = 0.0022 in the kidneys) and VY9-irrelevant peptide control group (*P* = 0.0274 in the liver, *P* = 0.0073 in the spleen and *P* = 0.0248 in the kidneys), respectively (Fig. 5). Notably, low levels of HTNV RNA loads in the organs were also displayed in Tg mice immunized with HTNV linear multi-epitope peptide PADRE-VV9. Specifically, the HTNV RNA load in the PADRE-VV9-inoculation Tg mice group was lower than that in the PBS-injection group in all the three organs (*P* = 0.0040 in the liver, *P* = 0.0043 in the spleen and *P* = 0.0279 in the kidneys) and also lower than that in the VY9-irrelevant peptide control group in the liver (*P* = 0.0140) and spleen (*P* = 0.0088) (Fig. 5). Importantly, lower levels of HTNV RNA loads were observed in the spleen of the Tg mice after immunization with PADRE-VV9 than in single epitope VV9-immunized mice group (*P* = 0.0360) (Fig. 5), indicating that immunization of HTNV multi-epitope peptide may provide better protection than that of single CTL epitope in spleen of the Tg mice.

## Discussion

In the current study, we designed the HTNV linear multi-epitope peptide consisting of HLA-A*02-restricted HTNV CTL epitope and PADRE, and evaluated the multi-epitope peptide as candidate vaccine in HLA-A2.1/K^b^ Tg mice as a virus infection model of immunizing the Tg mice following HTNV challenge. Compared with HTNV single VV9 epitope, linear multi-epitope peptide PADRE-VV9 could induce higher level of HTNV-specific CD8^+^ T-cell responses in Tg mice. Moreover, the HTNV-specific CTL responses induced by PADRE-VV9 was more effective than that of single VV9 epitope to reduce the HTNV RNA loads in Tg mice.These results may be important foundation for the development of novel peptide vaccines for HTNV.

It has been well established that severe or critical HFRS after HTNV infection is associated with decreased responses of CD4^+^ T-cells and CD8^+^ T-cells specific to multiple epitopes of HTNV [21, 22]. HTNV CTL epitope-specific T-cell responses have been proven to play a critical role in the control of virus infection through cytotoxic effects to clear the HTNV-infected cells directly. Given that T-cells are crucial for the control and clearance of HTNV infection, peptide vaccines inducing specific T-cell responses may offer promising approaches for the prevention of HTNV infection. The incorporation of CTL epitopes into the peptide vaccine could induce specific CTL responses, which may be more effective in the prevention of HTNV infection. As confirmed by our previous data, several HLA-A*02-restricted HTNV CTL epitope could induce protective CD8^+^ T-cell responses among HLA A*02^+^ HFRS individuals [21, 23]. However, low immunogenicity of the single epitope is a big obstacle in developing such peptide vaccines. A simple T-cell epitope is inadequate to act as an effective immunogen, leading to the requirement of much more complex antigens. Therefore, different approaches have been applied to make the epitope-based vaccines more potent and efficient [34, 35], one of which is to design linear multi-epitope peptides as candidate vaccines. However, it should be noted that T-cell epitopes in the vaccines would be restricted by the highly polymorphic HLA-I and II molecules.

In fact, HLA-A*02 allele is most frequently represented in the Chinese Han population [36]. Therefore, the multi-epitope peptides designed in this study, containing HLA-A*02-restricted HTNV-GP CTL epitope VV9, which has been proven to induce a high level of protective CD8^+^ T-cell responses in HFRS patients [23], will cover 30–40% of the at-risk population in China. The second component required for the design of the multi-epitope peptide vaccine was Th cell epitope, which could provide strong support to promote CTL response and a potent humoral response. A universal synthetic PADRE, which was originally developed by optimization of binding to HLA-DR, with high affinity to 15 out of 16 different HLA-DR molecules tested so far, was chosen for our multi-epitope peptide design [24, 37, 38]. PADRE can also bind with high to intermediate affinity to mouse I-A^b/d^ and I-E^b/d^ MHC haplotypes [38]. Therefore, PADRE peptide, used as a Th-epitope, was able to generate effective Th-cell responses, which could overcome the problems posed by the extreme polymorphism of HLA-DR molecules in the human population [24, 38]. Thus, our design of the HTNV linear multi-epitope peptide would allow for extensive coverage of the human population.

Additionally, tandem fusion of CTL epitopes and PADRE without proper linkers may result in the generation of junctional epitopes or a new protein. Therefore, short amino acid motifs (linkers) must be used between different epitopes connection. In our design, the epitopes were linked by an A-A-A linker contained in PADRE, which could play a principal role in retaining the structural features of each epitope. Moreover, the linkers are usually appropriate for proteasome-mediated cleavage or binding to a transporter associated with an antigen processing protein [39–41]. Therefore, the A-A-A linker may be helpful to achieve optimal processing for our multi-epitope peptides in vivo. The K-S-S at the amino-terminus of peptides was employed as an adaptor sequence, in which the lysine residue not only has a protective effect but also has the characteristic of forming connections easily with lipid components on the surface of the cell membrane, promoting the uptake of peptides by the antigen-presenting cells, then involved in T-cell induction following peptide immunization. The nature of the standard spacer S-S, positioned between the lysine and the epitope, was proven to be of low solubility and highly particulate, which is crucial for immunogenicity of the peptides [27, 37, 42].

Our previous studies indicated that protective immunity against HTNV involved strong epitope-specific CD8^+^ T-cell responses, promoting the clearance of the virus both in ex vivo HFRS patients and in vivo animal models [20, 21, 23]. In this study, the optimal immunization effects of the designed HTNV multi-epitope peptide in Tg mice were proven by the capacity of multi-epitope peptide to activate CD8^+^ T-cells responses. Evidence was initially shown from the finding that a much higher frequency of epitope VV9-specific CTLs was observed in the splenocytes of Tg mice stimulated with multi-epitope peptide than single VV9, as detected by IFN-γ ELISPOT assay. In line with the findings, multi-epitope peptide was also demonstrated to be capable of generating specific CTLs secreting granzyme B, demonstrating the potential cytotoxic effects of the specific CTLs induced by multi-epitope peptide. Meanwhile, expansion of VV9-specific CD8^+^ T-cells was observed from multi-epitope peptide-immunized Tg mice. The overall evidence suggested that multi-epitope peptide had good specificity and immunogenicity to induce specific-CD8^+^ T-cell response against HTNV antigens in vivo*.* Interestingly, there was a certain percentage of granzyme B^+^ CD8^+^ T-cells in unrelated VY9 peptide-immunized Tg mice and also in PBS-injection Tg mice, as shown in Fig. 3. Since there was peptide stimulation of the splenocytes from each Tg mice group, including PBS-immunized mice during the cell culture before granzyme B detection, we speculated that the peptide stimulation might be a driving factor for the CD8^+^ T-cell to produce granzyme B to some extent. Moreover, Arens R et al. showed that about 4% granzyme B^+^ CD8^+^ T-cells could be detected in wild-type C57BL/6 mice, which was similar to our findings and may prove that CD8^+^ T-cells in mice without any activation could also produce granzyme B [43]. However, the detection of CD107a, an important marker for the degranulation function of T cells, and the cytotoxic assay of the VV9-specific CD8^+^ T-cells in the immunized mice would be carried out in the future study, which may be more beneficial to prove the efficiency of CD8^+^ T-cell responses induced by HTNV linear multi-epitope peptide. Moreover, since the fluorescence intensity of CFSE will be weakened gradually with cell passages during the cell proliferation, the CFSE is widely used for cell proliferation assays. The wider peak with two small peaks shown in the PADRE-VV9-immunized mice after peptide stimulation indicated that the multi-epitope peptide had a good ability to stimulate specific CD8^+^ T-cell proliferation.

Notably, most of the susceptible animals to HTNV are rodents, however, HTNV-infected mice always carry HTNV in organs but without obvious symptoms after infection. The lack of an HFRS disease model that could recapitulate the features of human HFRS disease hampers the development of vaccines and the evaluation of post-exposure prophylactics, which may be a major shortcoming of the field. In the absence of a relevant disease animal model for HTNV infection, viral load reduction is considered as a more reliable indicator for HTNV vaccine efficacy in Tg mice [20, 23]. In this study, the effects of multi-epitope peptide-induced immune responses were evaluated in HLA-A2.1/K^b^ Tg mice with HTNV challenge as an infection model. The immunization of HTNV-inactivated vaccine in Tg mice was used as a positive control in our study, which has been proven to boost humoral responses to inhibit virus replication. Furthermore, the immunological mechanisms for viral clearance are also closely related with T-cell-mediated immune responses. The reduction in the levels of HTNV RNA loads in the liver, spleen and kidneys of Tg mice immunized with HTNV multi-epitope peptide could provide direct evidence to support that an effective CTL immunity could contribute to the clean of HTNV infection. The specific CTL responses induced by multi-epitope peptide immunization may also be considered as an important mechanism of immunoprotection in Tg mice. In this study, we selected peptide VV9 on HTNV-GP as CTL epitope to construct the multi-epitope vaccine. Although epitope VV9 has been proven to bind with HLA-A*02 with highest affinity among the seven HLA-A*02-restricted HTNV-GP CTL epitope, the HLA-A*02-restricted epitope LL9 (aa358-aa366, LIWTGMIDL) on HTNV-GP may be another appropriate candidate epitope for multi-epitope vaccine construction, given that epitope LL9 could induce higher frequencies of specific CTL responses and reduce the level of HTNV RNA loads in HTNV infected mice more effectively compared with that of epitope VV9. Therefore, we will try to research on epitope LL9 and explore the value and possibility of LL9 as a better candidate epitope for multi-epitope peptide vaccine construction in the future study.

Generally, there was a post-vaccination increase in VV9-specific CD8^+^ T-cell levels from Tg mice immunized with the multi-epitope peptide containing PADRE. Thus, it seems that the Th-cell epitope PADRE promoted the production of specific CTL responses. In fact, an important factor in a strong anti-viral approach is effective activation of CD4^+^ T-cell response, which is necessary for activation of CD8^+^ T-cell and generation of memory T-cells [44]. The success of linear multi-epitope peptide to elicit a potent CTL response against HTNV infection may be attributed to the existence of both CTL epitope and Th epitope, indicating that PADRE was critical to improving multi-epitope peptide vaccine-induced immune responses. However, a future study of CD4^+^ T-cell responses is necessary and essential to better evaluate the efficacy of the HTNV multi-epitope peptide vaccines.

## Conclusions

The present study demonstrated that the designed HTNV linear multi-epitope peptide vaccine consisting of HLA-A*02-restricted HTNV CTL epitope and CD4^+^ T-cell activator PADRE, could induce protective CD8^+^ T-cell responses to reduce HTNV RNA loads in HLA-A2.1/K^b^ Tg mice. The presence of PADRE may enhance the immunogenicity and efficiency of the multi-epitope peptide vaccine, which may provide some important information for the development of novel HTNV peptide vaccines. Additional studies are expected to confirm the effectiveness of the multi-epitope peptide vaccine in humans in the future.

## Data Availability

All data generated or analyzed during this study are included in this published article.

## Abbreviations

Ab: Antibody
AEC: 3-Amino-9-ethylcarbazole
ANDV: Andes virus
CFSE: 5,6-Carboxyfluorescein succinimidyl ester
CTL: Cytotoxic T cell
DOBV: Dobrava virus
ELISPOT: Enzyme-linked immunospot
FDA: Food and Drug Administration
GP: Glycoprotein
gp96: Glycoprotein 96
HCPS: Hantavirus cardiopulmonary syndrome
HFRS: Hemorrhagic fever with renal syndrome
HPLC: High-performance liquid chromatography
HTNV: Hantaan virus
IFN: Interferon
NP: Nucleocapsid protein
PADRE: Pan HLA-DR-binding epitope
PBS: Phosphate-buffered saline
PE: Phycoerythrin
PHA: Phytohemagglutinin
PUUV: Puumala virus
SEM: Standard error of mean
SEOV: Seoul virus
SFC: Spot-forming cell
SNV: Sin Nombre virus
Tg: Transgenic
Th: T helper
VV9: VMASLVWPV
VY9: VPILLKALY
